# Aligning implementation and user-centered design strategies to enhance the impact of health services: results from a concept mapping study

**DOI:** 10.1186/s43058-020-00020-w

**Published:** 2020-02-26

**Authors:** Alex R. Dopp, Kathryn E. Parisi, Sean A. Munson, Aaron R. Lyon

**Affiliations:** 1 Department of Psychological Science, University of Arkansas, 216 Memorial Hall, Fayetteville, AR 72701 USA; 2 Department of Behavioral and Policy Sciences, RAND Corporation, 1776 Main Street, Santa Monica, CA 90401 USA; 3 Department of Human Centered Design and Engineering, University of Washington, 3960 Benton Lane NE, 428 Sieg Hall, Seattle, WA 98195 USA; 4 Department of Psychiatry and Behavioral Sciences, School of Medicine, University of Washington, 1959 NE Pacific Street Box 356560, Room BB1644, Seattle, WA 98195 USA

**Keywords:** Implementation strategies, User-centered design, Human-centered design, Concept mapping, Evidence-based practice

## Abstract

**Background:**

Innovative approaches are needed to maximize fit between the characteristics of evidence-based practices (EBPs), implementation strategies that support EBP use, and contexts in which EBPs are implemented. Standard approaches to implementation offer few ways to address such issues of fit. We characterized the potential for collaboration with experts from a relevant complementary approach, user-centered design (UCD), to increase successful implementation.

**Method:**

Using purposive and snowball sampling, we recruited 56 experts in implementation (*n* = 34) or UCD (*n* = 22). Participants had 5+ years of professional experience (*M* = 10.31), worked across many settings (e.g., healthcare, education, human services), and were mostly female (59%) and white (73%). Each participant completed a web-based concept mapping structured conceptualization task. They sorted strategies from established compilations for implementation (36 strategies) and UCD (30 strategies) into distinct clusters, then rated the importance and feasibility of each strategy.

**Results:**

We used multidimensional scaling techniques to examine patterns in the sorting of strategies. Based on conceptual clarity and fit with established implementation frameworks, we selected a final set of 10 clusters (i.e., groups of strategies), with five implementation-only clusters, two UCD-only clusters, and three trans-discipline clusters. The highest-priority activities (i.e., above-average importance and feasibility) were the trans-discipline clusters plus facilitate change and monitor change. Implementation and UCD experts sorted strategies into similar clusters, but each gave higher importance and feasibility ratings to strategies/clusters from their own discipline.

**Conclusions:**

In this concept mapping study, experts in implementation and UCD had perspectives that both converged (e.g., trans-discipline clusters, which were all rated as high-priority) and diverged (e.g., in importance/feasibility ratings). The results provide a shared understanding of the alignment between implementation science and UCD, which can increase the impact and sustainability of EBP implementation efforts. Implications for improved collaboration among implementation and UCD experts are discussed.

Contributions to the literature

- Successful implementation of evidence-based practices (EBPs) requires innovative strategies that can improve the fit between those practices and their implementation contexts. User-centered design (UCD) offers a set of such strategies, many of which are not known to implementation scientists.
- Through a structured conceptualization exercise, a multidisciplinary panel of experts indicated that many strategies used by implementation and UCD experts are complementary (i.e., single-discipline clusters of strategies) but also identified trans-discipline clusters that represent key points of alignment.
- The findings deepen our understanding of how multidisciplinary experts might collaborate to apply implementation and UCD strategies toward improved use of EBPs.

## Background

Implementation science—an interdisciplinary field in the health sciences that is focused on improving the use of research evidence in everyday practice settings—has long focused on promoting the use of evidence-based practices (EBPs) for the assessment of, intervention with, and management of medical and behavioral health conditions. Unfortunately, even when implementation occurs, EBPs typically show reduced impacts in community settings and are rarely sustained once implementation support ends [1, 2]. Numerous characteristics of EBPs—and the strategies used to support their implementation—can undermine their effectiveness in typical health service settings by producing a mismatch with the real-world needs of providers, patients, and service organizations (see [3] for a review). Examples of design problems include low ease of use (e.g., interventions that lack flexibility needed for community patient populations), high complexity (e.g., screening tools that are difficult for providers to administer and interpret correctly), and incompatibility with constraints of the delivery setting (e.g., time-intensive training and consultation models for implementation). To maximize the public health benefits of applying research evidence, implementation efforts will require complementary approaches that can enhance EBP fit with the contexts in which they are implemented [4–6]. In the present study, we sought to characterize the potential of one such approach, user-centered design, to provide a set of strategies that can align with implementation strategies to better support EBPs’ use in community settings.

### Ongoing challenges in promoting implementation success

Over the past several decades, experts in implementation research and practice have identified a number of promising strategies for the implementation of EBPs. The most comprehensive review of these strategies is the Expert Recommendations for Implementing Change (ERIC) study, in which a panel of 35 implementation experts defined 73 discrete implementation strategies through a Delphi consensus-building process that expanded on the results of an earlier systematic review [7] and then sorted those strategies into nine conceptually distinct categories while also rating their importance and feasibility [8]. The ERIC study provided a much-needed common language and set of best-practice strategies used in implementation research and practice. However, a close examination of the strategies reveals important gaps in the approach currently taken by the field. For example, Dopp and colleagues [9] examined the ERIC compilation using the multilevel domains specified in the Consolidated Framework for Implementation Research (CFIR [10];) and found that most of the 73 strategies focus on changes in the individuals and systems (inner/outer setting) that will adopt a health services innovation, whereas only three seemed to address the possibility of tailoring the innovation to local contexts (i.e., “develop and implement tools for quality monitoring,” “develop educational materials,” and “promote adaptability”). Given that EBP usability is a key upstream determinant of implementation outcomes such as acceptability, appropriateness, and feasibility [11], as well as findings that context-specific modifications to EBPs are common and influential during implementation efforts [2, 12–14], current approaches to the promotion of implementation success are likely to be incomplete.

Recently, researchers have observed that both EBPs and implementation strategies have fundamental design problems that limit their effectiveness in diverse health service settings [3]. Health care providers and other stakeholders (e.g., patients, administrators) often encounter significant usability challenges with EBPs, both in terms of the tasks involved (e.g., clinical techniques, goal setting, practice-specific supervision) and the packaging that structures the tasks (e.g., manuals, worksheets, length and modality of sessions). Although some of these challenges could be addressed through improved attention to design during initial development of EBPs, scholars have increasingly argued that EBPs are frequently “over-designed” in research settings—leading to inclusion of features that are not necessary or useful to end users—and instead recommended that health care practices be optimized within their ultimate implementation setting [11, 15]. Recognizing that the ERIC [7] compilation, while groundbreaking, speaks only sparingly to aspects of EBP design that may improve uptake, we suggest that there is a need for additional strategies that attend directly to those issues of design. To that end, it may be useful to seek innovative strategies from outside the health service fields and deepen our understanding of how multidisciplinary experts might collaborate to apply those strategies.

### Potential of user-centered design

The field of user-centered design (UCD) holds considerable potential for increasing the impact and sustainment of EBPs (see [3, 11, 16, 17]). Drawing from research in human–computer interaction, user experience design, service design, and cognitive psychology, UCD and the closely-related field of human-centered design offer a set of principles and strategies that guide the design of an innovation from the perspectives of (and with input from) the people who will ultimately use that innovation [18–21]. Dopp and colleagues recently published a glossary of 30 UCD strategies for implementation researchers [22]; illustrative examples include identification of users and user needs, cycles of rapid prototyping and iterative development, co-creation and usability testing sessions with users, and interpretation sessions with stakeholders. In contrast to the ERIC implementation strategies, far more UCD strategies targeted the innovation (33%) or individuals (40%) involved in the implementation effort, although UCD can also be used to modify the inter- or intra-organizational context to better suit an EBP [22]. The ultimate aim of UCD is to make innovations and systems “useable and useful” for specified users, activities, and goals [23]. UCD can be applied to the development and improvement of digital and analog technologies (e.g., [24]), service systems (e.g., [25]), and training processes (e.g., [26]). It most frequently has been used to design new health services and technologies (e.g., [17, 27, 28]), whereas applications to the delivery and sustainment of already-designed EBPs (including the design of implementation strategies) remain rare. Health service fields like implementation science have yet to apply UCD extensively, although there are a growing number of examples for both intervention design studies (e.g., [29, 30]) and conceptual models (e.g., [15, 31]). Findings to date suggest that UCD has high relevance to most (if not all) EBPs, implementation strategies, and practice contexts within health care (see [31] in particular).

Despite its potential, it remains unclear how UCD fits within the evolving landscape of implementation research and practice. Implementation is already a highly interdisciplinary field and new collaborations between implementation experts and UCD experts will be essential to capitalize on the promise of UCD for health services. Experts from these two fields have only recently begun joining together to examine the role of design in implementation, and their efforts have been primarily in the form of conceptual frameworks (e.g., [15, 31]). As a step toward better understanding the alignment of implementation and UCD strategies, we used concept mapping [32] to characterize how experts from each discipline conceptualize the relations among the strategies described in these frameworks. Our study offers a novel empirical understanding of the proposed conceptual relationship between these two disciplines.

## Method

The method for this study was previously described in a published study protocol [9]. Herein we summarize the method and provide additional details about its actual execution, but readers should refer to [9] for a more fully detailed description. Additional file 1 contains a checklist of reporting guidelines for mixed-method research (supplemented with specific items for concept mapping) that we completed for the study.

### Recruitment and participants

To ensure our participants had appropriate expertise and constituted an internationally representative sample, recruitment used a combination of purposive and snowball sampling [33] in which we sent invitation emails to experts in implementation and/or UCD. Purposive sampling targeted experts from research centers and professional organizations that were centers of excellence for research in implementation and/or UCD; snowball sampling involved nominations from participants who completed the study. Interested participants contacted the study coordinator (second author) and were given login information for Concept Systems Global MAX (CSGM [34];), the web-based software platform that we used to conduct concept mapping. Once they logged into CSGM, the participants read and electronically signed the informed consent form, completed a short demographic questionnaire, and then began the concept mapping exercise.

The 56 participants were implementation experts (*n* = 34; 61%) and UCD experts (*n* = 22; 39%). Expertise was self-reported based on experience in research, practice/industry, and/or education over the past 5 or more years. We did not ask participants to identify specific areas of expertise, but we believe many had both research and applied experience in their discipline based on our recruitment methods and our interactions with participants during the study. Participants averaged 10.3 years of professional experience (SD = 6.7, range = 5–35). When asked how often their work involved interdisciplinary collaboration, half of participants indicated 80–100% of the time (top fifth), with increasingly smaller proportions endorsing 61–80%, 41–60%, 21–40%, and 0–20% of the time (21%, 16%, 11%, and 2% endorsement, respectively). Most participants (88%) reported focusing on health care in their work, but many also reported working with the prevention and health promotion (36%), education (18%), or human services (e.g., justice, child welfare, housing) (16%) sectors. When asked which CFIR domains they seek to improve through their work, most participants endorsed the individual (88%) and intervention/innovation (84%) levels, a smaller majority indicated the inner setting (70%), and the smallest proportion indicated the outer setting (34%). Finally, because the concept mapping software program limited the number of demographic questions that we could ask participants, we collected gender and race data in a follow-up Qualtrics survey which was completed by 51 participants (9% missing). Demographic data indicated that the sample was 59% female (*n* = 33; another 18 [32%] were male) and 73% white (*n* = 41; another six [11%] were Asian and the remaining four [8%] were other races).

We originally aimed to recruit 30 experts from each discipline [9], but more participants self-reported expertise in implementation than anticipated at enrollment (which filled slots originally intended for UCD experts), and several recruited UCD experts did not complete the study. Nevertheless, our sample size was still adequate for concept mapping as it exceeded the recommended sample size of *n* ≥ 15 per group [35].

### Procedures

#### Concept mapping

We used concept mapping [32] to systematically capture the relationships that participants perceived between different concepts or ideas (i.e., implementation strategies and UCD strategies). This method guides participants through a structured conceptualization process where they sort ideas into related groups and then rate the ideas on key dimensions. It is a self-contained mixed-method approach (i.e., incorporating both qualitative and quantitative data collection and analysis approaches) consisting of four phases: (1) idea generation, (2) sorting, (3) rating, and (4) analysis.

- **Idea generation.** As detailed in [9], our research team generated the ideas/concepts for participants to sort and rate by using existing resources that documented implementation and UCD strategies. For implementation, we selected a subset of 36 strategies from the full list of ERIC [7] strategies, with strategies chosen to maximize representativeness across (i) CFIR domains, (ii) categories of implementation strategies from a previous concept mapping study [8], and (iii) importance ratings (also collected by [8]). For UCD, we included all 30 strategies from our aforementioned compilation [22]. We uploaded each strategy (name and brief definition) into CSGM as a separate “statement” for subsequent sorting and rating by participants.
- **Sorting and rating.** The middle two phases of concept mapping, sorting and rating, were completed in tandem through the CSGM platform. CSGM allows participants to complete tasks in any order, and participants could also stop and start the activities as often as they wished. Our instructions and rating dimensions were adapted from ERIC [8].

For the sorting step, participants sorted each of the 66 implementation and UCD strategies into groups based on their view of the strategies’ meaning or theme. The order of strategy presentation was randomized, with no distinction between implementation versus UCD strategies. For the rating step, participants rated each strategy on its importance and feasibility on a scale ranging from 1 (least important/feasible) to 5 (most important/feasible). Ratings for importance and feasibility were completed separately.

#### Post-survey

After participants completed all steps in CSGM, the system displayed a link to the post-survey in Qualtrics which collected additional demographic information; questions about challenges in collaboration between implementation experts and UCD experts (which were not yet analyzed for this initial study); and snowball sampling nominations. Upon completion, participants received a unique link for a $20 electronic gift card.

### Analytic strategy

The final step of concept mapping, data analysis [32], involved using multidimensional scaling techniques (embedded in CSGM [34]) to identify clusters of implementation and UCD strategies that were generated most consistently across participants. We retained and analyzed data provided by all participants, including those who did not complete all study steps, although usable data were available from most participants (98% for sorting; 96% for rating).

CSGM can empirically generate any number of clusters, so the research team reviewed the results for conceptual clarity and credibility before selecting which set of clusters to report. To guide our thinking, we examined cluster maps produced by CSGM, which represent the relatedness of concepts within and between clusters in terms of visual distance. We also considered the extent to which clusters were consistent with or expanded upon the (1) clusters of implementation strategies identified in the ERIC study [8]; (2) CFIR domains [10]; and (3) the Integrated Promoting Action on Research Implementation in Health Services (i-PARIHS) framework [36], which describes the process of facilitating EBP use in practice settings by attending to characteristics of the EBP, recipients, and context (i-PARIHS is a process framework, which complements the determinant-focused nature of CFIR [37]). We began with a 13-cluster solution, which is one SD above the mean number of clusters in a typical concept mapping solution [35], and examined splitting and merging of clusters in a stepwise fashion. Once we selected the final set of clusters, we calculated average importance and feasibility ratings for each cluster and strategy. We used unweighted averages because weighting by subsample size (to account for the different number of implementation vs. UCD experts in the sample) resulted in very small changes to the average values, with no changes to study conclusions. We also examined ladder graphs, which provide a visual representation of the relationship between dimensions (e.g., importance and feasibility) within and across clusters. In addition, we explored the number and types (i.e., by discipline) of strategies in each cluster.

Following the initial analyses of concept mapping data from all participants, we also examined results separately by subgroup (i.e., implementation vs. UCD experts). We applied the same analytic approach described previously with data separated by discipline, and we evaluated whether there were observed differences in the number, content, or ratings of the clusters. We also used multivariate general linear models to test for differences in ratings of each cluster’s perceived importance and feasibility across disciplines.

## Results

### Cluster solution

The stress value for the multidimensional scaling analysis of our data was 0.188, well below the 0.365 cutoff recommended for ensuring adequate consistency among respondents [32], which indicated that we could proceed with identifying a cluster solution. After examining and discussing solutions that ranged from 13 down to 8 clusters over a period of several weeks, we identified a 10-cluster solution. The research team unanimously agreed that this solution offered the greatest conceptual clarity and contained concepts that aligned with the ERIC cluster solution [8] and relevant implementation frameworks [10, 36]. We also followed the process and guidelines outlined by the ERIC team [8] to achieve consensus on labels for the final clusters.

**Fig. 1:**
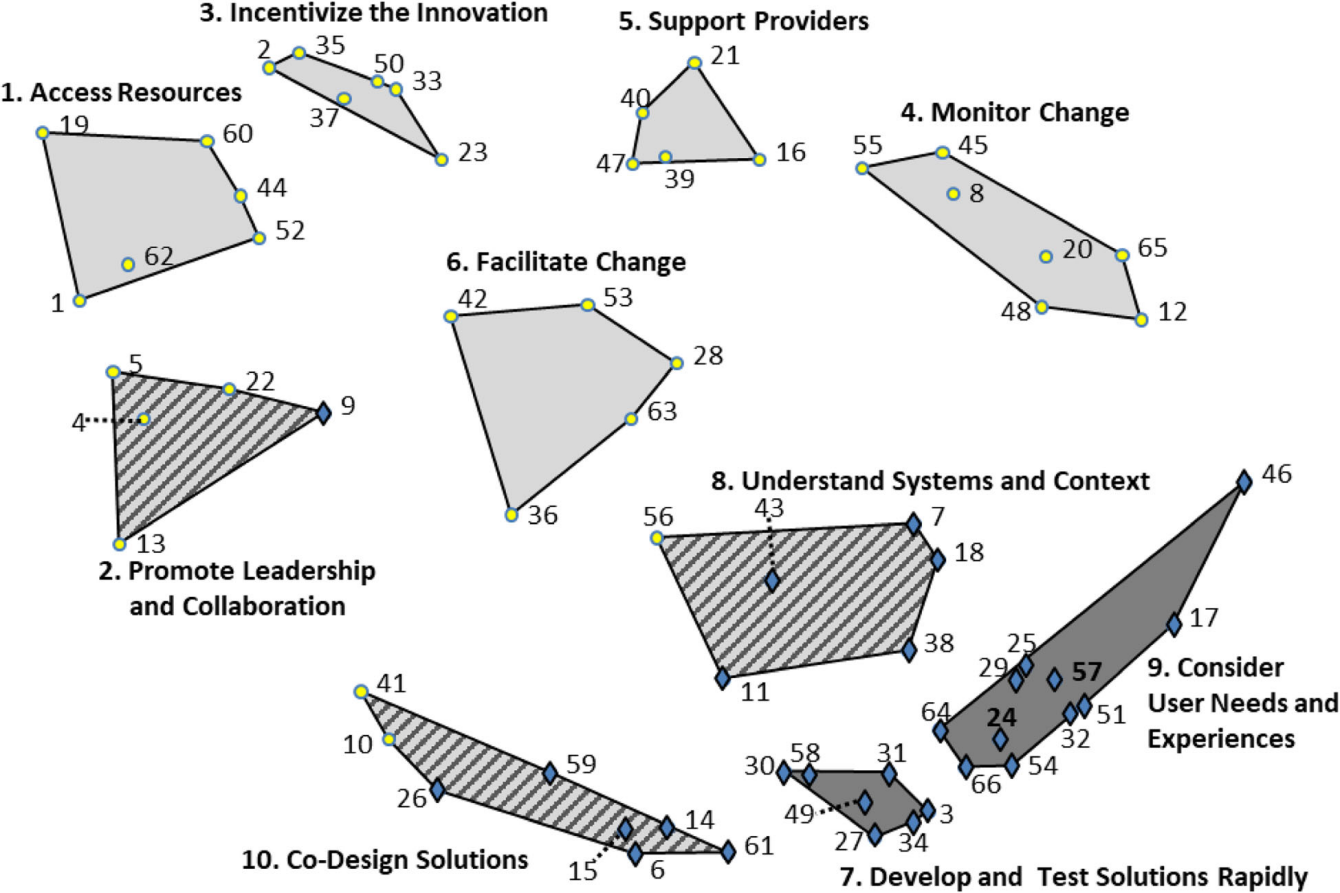
Cluster map of implementation and user-centered design (UCD) strategies. The map reflects the product of an expert panel (valid response *n* = 55) sorting 66 discrete strategies into groupings by similarity. Circles indicate implementation strategies and diamonds indicate UCD strategies. The number accompanying each strategy allows for cross-referencing to the list of strategies in Table 1. Light-colored clusters are comprised entirely of implementation strategies; dark-colored clusters are comprised entirely of UCD strategies; and multi-colored clusters are comprised of strategies from both disciplines. Spatial distances reflect how frequently the strategies were sorted together as similar. These spatial relationships are relative to the sorting data obtained in this study, and distances do not reflect an absolute relationship

**Fig. 2:**
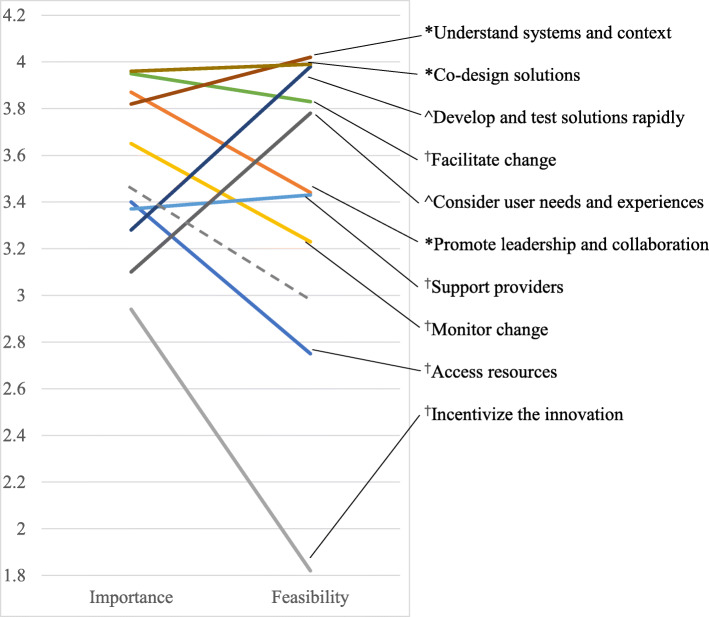
Ladder graph of the average importance and feasibility ratings for the cluster solution (see Fig. 1). The graph reflects the product of an expert panel (valid response *n* = 54) rating 66 discrete implementation and user-centered design (UCD) strategies on a scale from 1 to 5. The range of values on the *y*-axis reflect the mean rating obtained for each cluster (as reported in Table 1) with a color-coded line joining the importance and feasibility ratings for each cluster. The cluster names are listed to the right with a line indicating the respective part of the graph for that cluster’s ratings († = implementation-only cluster, ^ = UCD-only cluster, * = trans-discipline cluster). The gray dotted line indicates the average importance (3.45) and feasibility (2.92) ratings across all strategies; clusters that fall fully above this line on the ladder graph were considered “high-priority”

Figure 1 presents a cluster map that visually represents the relationships among the 66 strategies, with symbols on the map representing implementation strategies (circles) or UCD strategies (diamonds). Table 1 presents a complete list of strategies, organized by cluster, and summarizes the characteristics of the strategies and clusters. Five clusters were comprised entirely of implementation strategies, two were comprised entirely of UCD strategies, and the remaining three clusters contained strategies from both disciplines. Average importance ratings ranged from 2.4 to 4.5 for individual strategies and from 2.9 to 4.0 for clusters. Average feasibility ratings ranged from 1.5 to 4.5 and from 1.8 to 4.0 for strategies and clusters, respectively. Importance and feasibility ratings were highly correlated (*r* = 0.57). Figure 2 presents a ladder graph that visually represents the importance and feasibility ratings of each cluster. We considered clusters that fell above the mean on both sides of the ladder graph to be “high-priority” because they were highly important and feasible. All three of the trans-disciplinary clusters were high-priority, as were two clusters of implementation strategies.

**Table 1:** Summary of strategies and clusters, including key characteristics

Cluster/strategy		Importance^a^	Feasibility^b^	Discipline	Alternate UCD cluster^c^
1. Access resources		3.4	2.8	100% IMP	n/a
1	Work with educational institutions	3.2	3.1	IMP	–
19	Fund and contract for the clinical innovation	3.7	2.0	IMP	–
44	Mandate change	3.2	2.8	IMP	–
52	Develop resource sharing agreements	3.1	2.9	IMP	–
60	Access new funding	3.8	2.2	IMP	–
62	Use train-the-trainer strategies	3.4	3.5	IMP	–
2. Promote leadership and collaboration		3.9	3.4	80% IMP 20% UCD	n/a
4	Identify and prepare champions	4.2	4.0	IMP	–
5	Recruit, design, and train for leadership	4.1	3.7	IMP	–
13	Build a coalition	4.1	3.7	IMP	–
22	Obtain formal commitments	3.2	3.5	IMP	–
9	Build a user-centered organizational culture	3.7	2.3	UCD	–
3. Incentivize the innovation		2.9	1.8	100% IMP	n/a
2	Place innovation on fee for service lists/formularies	3.5	2.2	IMP	–
23	Increase demand	2.8	1.9	IMP	–
33	Change accreditation or membership requirements	2.4	1.6	IMP	–
35	Alter patient/consumer fees	2.9	1.7	IMP	–
37	Alter incentive/allowance structures	3.5	2.2	IMP	–
50	Create or change credentialing and/or licensure standards	2.6	1.5	IMP	–
4. Monitor change		3.7	3.2	100% IMP	n/a
8	Change record systems	3.4	2.5	IMP	–
12	Purposefully reexamine the implementation	4.1	4.0	IMP	–
20	Develop and implement tools for quality monitoring	3.8	3.1	IMP	–
45	Audit and provide feedback	3.9	3.6	IMP	–
48	Use data experts	3.0	3.3	IMP	–
55	Facilitate relay of clinical data to providers	3.8	2.9	IMP	–
65	Develop and organize quality monitoring systems	3.6	3.2	IMP	–
5. Support providers		3.4	3.4	100% IMP	n/a
16	Remind clinicians	2.8	3.6	IMP	–
21	Conduct ongoing training	3.7	3.6	IMP	–
39	Centralize technical assistance	3.1	3.0	IMP	–
40	Provide ongoing consultation	3.7	3.7	IMP	–
47	Provide local technical assistance	3.6	3.3	IMP	–
6. Facilitate change		4.0	3.8	100% IMP	n/a
28	Tailor strategies	4.3	3.9	IMP	–
36	Facilitation	3.7	3.7	IMP	7
42	Organize clinician implementation team meetings	3.7	3.6	IMP	–
53	Develop educational materials	3.6	4.4	IMP	–
63	Promote adaptability	4.4	3.7	IMP	–
7. Develop and test solutions rapidly		3.3	4.0	100% UCD	
3	Use generative object-based techniques	3.0	3.9	UCD	6
27	Engage in cycles of rapid prototyping	3.9	3.9	UCD	6
30	Conduct focus groups about user perspectives	3.4	4.5	UCD	9
31	Use associative object-based techniques	2.4	3.8	UCD	9
34	Engage in live prototyping	3.7	3.6	UCD	6
49	Conduct interviews about user perspectives	3.7	4.5	UCD	9
58	Develop personas and schemas	2.9	3.9	UCD	9
8. Understand systems and context		3.8	4.0	83% UCD 17% IMP	
7	Define work flows	3.8	4.2	UCD	8
11	Engage in iterative development	4.5	3.9	UCD	7
18	Apply process maps to systems-level behavior	3.1	3.5	UCD	8
38	Conduct observational field visits	4.3	4.1	UCD	9
43	Prepare and present user research reports	3.3	4.3	UCD	8
56	Assess for readiness and identify barriers and facilitators	3.9	4.1	IMP	8
9. Consider user needs and experiences		3.1	3.8	100% UCD	
17	Conduct experience sampling	2.7	3.3	UCD	9
24	Conduct usability tests	4.0	4.2	UCD	6
25	Apply task analysis to user behavior	3.3	3.9	UCD	9
29	Develop a user research plan	3.7	4.1	UCD	6
32	Conduct heuristic evaluation	2.4	3.7	UCD	6
46	Examine automatically generated data	3.4	3.8	UCD	7
51	Conduct artifact analysis	2.8	3.6	UCD	9
54	Conduct competitive user experience research	3.0	3.6	UCD	9
57	Develop experience models	3.1	3.7	UCD	9
64	Collect quantitative survey data on potential users	3.1	4.1	UCD	9
66	Use dialogic object-based techniques	2.8	3.7	UCD	9
10. Co-design solutions		4.0	4.0	75% UCD 25% IMP	
6	Conduct co-creation sessions	4.1	3.9	UCD	6
14	Recruit potential users	4.1	4.1	UCD	9
15	Conduct design charrette sessions with stakeholders	3.1	3.6	UCD	6
26	Conduct interpretation sessions with stakeholders	3.7	4.0	UCD	8
59	Design in teams	3.9	4.1	UCD	7
61	Define target users and their needs	4.5	4.4	UCD	6
10	Conduct local consensus discussions	4.2	3.8	IMP	8
41	Involve patients/consumers and family members	4.2	4.0	IMP	8

Strategies are organized by discipline (*IMP* implementation, *UCD* user-centered design) within each cluster

^a^Rating scale ranged from 1 (relatively unimportant) to 5 (extremely important)

^b^Rating scale ranged from 1 (not at all feasible) to 5 (extremely feasible)

^c^For clusters dominated by UCD strategies, indicates the alternate cluster in which a given strategy was located based on a nine-cluster solution from sorting responses of UCD expert participants (valid response *n* = 21); those clusters (detailed in Additional file 2) are as follows: 6. Develop and test solutions rapidly; 7. Unnamed new cluster; 8. Understand systems and context; and 9. Consider user needs and experiences

### Comparison of results by discipline

We were able to examine cluster solutions separately by discipline, given that we found adequate stress values for the implementation expert data (0.200) and UCD expert data (0.251). However, the research team determined that the discipline-specific cluster solutions did not differ in meaningful ways from the primary 10-cluster solution, with one exception: UCD experts sorted UCD strategies somewhat differently from implementation experts, producing a 9-cluster solution that replaced one of the four UCD-dominant clusters (co-design) with a difficult-to-interpret cluster that contained several key approaches to the design process (e.g., iterative development, design in teams) as well as implementation facilitation. The other three UCD-dominant clusters in this alternate solution were all conceptually similar to those from the primary cluster solution—to the point that we retained the same names—but the makeup of strategies within those clusters differed by 43–67%. The alternate solution did not offer any major conceptual or practical advantages over the primary cluster solution (indeed, we were unable to agree on a name for the new cluster), so we focused on the primary cluster solution for our remaining analyses. For consideration, however, we present the cluster map of the four alternate UCD-dominant clusters in Additional file 2, and we indicate in Table 1 the alternate cluster assignment for each strategy from the UCD-dominant clusters.

Next, for the primary 10-cluster solution, we compared average cluster ratings between disciplines. Multivariate general linear models indicated that there were significant differences between implementation and UCD experts’ ratings of importance (*F*_10,43_ = 5.12, *p* < 0.001, with significant differences for 5 individual clusters) and feasibility (*F*_10,43_ = 5.78, *p* < 0.001, with significant differences for 7 individual clusters). A *post hoc* repeated-measures analysis confirmed that these differences in cluster ratings were driven by participants’ tendency to rate strategies from their own discipline as more important and more feasible (*F*_2,50_ = 20.56, *p* < 0.001). Table 2 presents the importance and feasibility ratings of each cluster by implementation versus UCD experts. The table also reports, from the multivariate models, the statistical significance and magnitude (calculated using Cohen’s *d* effect sizes) of all between-discipline differences in ratings for each cluster. Whenever the difference between disciplines was significant, the higher ratings came from the same discipline as the majority of strategies in the cluster. The magnitude of differences fell within the small to medium range (0.2 < *d*s < 0.8). Despite these differences, the high-priority clusters had high ratings on importance and feasibility (with small to negligible differences, *d*s < 0.5) across disciplines.

**Table 2:** Average cluster ratings compared between disciplines

Cluster	Importance			Feasibility		
	IMP experts	UCD experts	*d*^a^	IMP experts	UCD experts	*d*^a^
1. Access resources	3.5	3.2	0.18	2.9	2.5	0.35*
2. Promote leadership and collaboration	3.9	3.8	0.05	3.5	3.3	0.17
3. Incentivize the innovation	3.2	2.6	0.28*	2.0	1.6	0.25
4. Monitor change	3.8	3.4	0.31*	3.4	3.0	0.33*
5. Support providers	3.6	2.9	0.50*	3.8	2.9	0.68*
6. Facilitate change	4.0	3.8	0.19	4.0	3.6	0.28*
7. Develop and test solutions rapidly	3.0	3.7	− 0.43*	3.7	4.4	− 0.65*
8. Understand systems and context	3.8	3.8	− 0.01	4.0	4.1	− 0.11
9. Consider user needs and experiences	3.0	3.3	− 0.27	3.5	4.1	− 0.50*
10. Co-design solutions	3.8	4.1	− 0.28*	3.8	4.2	− 0.32*

Importance and feasibility values reflect the product of an expert panel (valid response *n* = 54) rating 66 discrete implementation and user-centered design strategies on a scale from 1 to 5. Comparisons based on *F*_10,43_ multivariate tests; * = *p* < 0.05

*IMP experts* implementation experts, *UCD experts* user-centered design experts

^a^Cohen’s *d* effect size, also known as the standardized mean difference; calculated such that positive values reflect higher ratings by implementation experts and negative values reflect higher ratings by UCD experts; thresholds are *d* = 0.2 for small effect, *d* = 0.5 for medium effect, and *d* = 0.8 for large effect

We had originally planned [9] to examine differences between professions in the number and types of strategies in the identified clusters using *χ*^2^ analysis. Since we arrived at a common cluster solution for both disciplines, however, we deemed such an analysis unnecessary.

## Discussion

Implementation researchers who wish to increase the public health impact of EBPs need to consider novel approaches, such as UCD, that can improve the fit between EBPs, the strategies used to implement them, and their implementation contexts. This concept mapping study explored 56 experts’ perspectives on the potential interdisciplinary convergence and alignment among implementation strategies and UCD strategies. Based on their input, we identified 10 clusters of strategies (5 implementation-only, 2 UCD-only, and 3 trans-discipline) that deepen our understanding of how UCD strategies relate to traditional strategies for supporting implementation efforts. Given this observed clustering of strategies, we conclude that implementation science and UCD offer complementary approaches to improving health and well-being, with each discipline making unique contributions that could be strengthened by the other. This represents less interdisciplinary overlap than we had anticipated when planning the study, given the common objectives of the two fields (i.e., we referred to “integrating” implementation and UCD strategies in our protocol [9]), and demonstrates the value of using empirical methods to inform conceptualization and confirm (or disconfirm) impressions. Of course, as a preliminary study, this one also had limitations (e.g., the lower than anticipated recruitment of UCD experts) and left many unanswered questions, so we highlight the need for additional research throughout our subsequent discussion.

The potential for collaboration between implementation and UCD experts is most evident in the three trans-discipline clusters of strategies, which experts from both disciplines rated above-average on both importance and feasibility. This suggests that implementation and UCD experts may be most ready to align around the activities represented by these clusters in ways that produce mutual benefit. For example, UCD offers specific tools and methods that can help implementation experts achieve important aims such as identifying barriers and facilitators to change [38] (located in cluster 8, “understand systems and context”) and co-designing solutions with stakeholders (cluster 10) [39]. Through collaboration, implementation experts could incorporate more effective and feasible ways to achieve their aims while UCD experts may benefit from increased opportunities to apply their expertise to pressing, large-scale health needs. The final trans-discipline cluster, “promote leadership and collaboration,” differs in that it is dominated by implementation strategies, but UCD contributes the strategy “build a user-centered organizational culture.” UCD experts may find that organization-focused strategies (such as “identify and prepare champions”) can help make a user-centered culture more feasible, while implementation experts might consider whether user-centeredness is an important dimension to address in existing leadership- and collaboration-oriented strategies (e.g., [40]). However, it is important to note that these results are at the cluster level; in future work, we plan to examine “go-zone” graphs [32] that plot individual strategies along key dimensions (e.g., importance vs. feasibility, implementation vs. UCD experts) to identify discrete strategies within and across clusters that are particularly promising for cross-disciplinary collaboration.

Most implementation and UCD strategies were located in distinct (rather than trans-discipline) clusters, which suggests an additional level of complementarity in that the two disciplines each contribute novel approaches to addressing common problems. In keeping with key implementation frameworks [10, 36], the expert panel identified clusters of implementation strategies that addressed intra- and inter-organizational contexts (“access resources,” “incentivize the innovation”), EBP providers (“support providers”), or the implementation process itself (“monitor change,” “facilitate change”). The latter two clusters were the remaining high-priority clusters, consistent with how the i-PARIHS framework [36] proposes facilitation as a key ingredient for successful implementation (CFIR [10] also includes a “process” domain but emphasizes it less). These observations provide validation of our cluster solution, even though the observed implementation-only clusters did not closely replicate ERIC clusters [8] (e.g., strategies from the ERIC cluster “utilize financial strategies” were split across “access resources” and “incentivize the innovation”). Rather than revealing some universal truth, cluster mapping instead represents how a group thinks about particular issues or ideas—so these differing conceptualizations are not problematic, as they were theoretically consistent and not directly contradictory. Of course, even implementation-specific clusters may still benefit from collaboration with UCD experts (e.g., by helping actualize effective strategies to “remind clinicians”), although the path forward may be less evident than in trans-disciplinary clusters.

The apparent context dependency of concept mapping solutions suggests a number of other future research directions. At the most basic level, it will be important to see how well other samples of implementation and UCD experts can replicate the observed cluster solution, especially across different subdomains in health care (e.g., medical vs. mental health, adults vs. pediatrics). More research is also needed to examine whether conceptualizations of these strategies differ among experts in the research versus practice of implementation and UCD, given that our recruitment strategy did not distinguish among these two types of expertise (between which there are notable gaps in both fields [41, 42]). Finally, the compilations from which we drew strategies for sorting and rating in this study [7, 22] are themselves context-dependent in that they primarily describe implementation and UCD activities within health care. A recent project adapted the ERIC implementation strategies for use in school settings [43] and replicated key importance and feasibility ratings for each strategy [44]; the findings showed that ratings for one-third of the strategies shifted meaningfully from the original ERIC compilation to the school-adapted set. Future research should similarly consider how UCD strategies transfer.

The two UCD-only clusters offer important extensions of the implementation strategies summarized above, as both offer approaches to address the often-overlooked innovation/intervention domain in implementation frameworks [10, 36]. These clusters were consistent with a separate framework for UCD [17] which proposes a cyclical process of identifying user needs (“consider user needs and experiences”), and then developing prototype solutions with which users interact (“develop and test solutions rapidly”). This rapid, iterative, and user-engaged approach to problem-solving is a key contribution that could help implementation experts achieve more rapid and pragmatic impact [45]—and again, UCD experts may also see their skills achieve broader-scale impact with complementary implementation strategies. The specifics of the conceptualization of UCD strategies within clusters remain less clear, as evidenced by the alternate clusters generated from the UCD experts’ data, but this may reflect the more nascent state of describing UCD strategies. Four researchers with expertise in implementation and UCD developed our UCD strategy compilation through a rapid literature review [22], whereas ERIC was based on a systematic literature review followed by a Delphi consensus-building exercise with 35 implementation experts [7]. Like implementation science, UCD is a diverse, innovative field that remains highly variable in terms of language and approaches, yet to date, UCD has focused less on consistently categorizing its own processes. Therefore, more research may be needed to achieve a compilation of UCD strategies that fully represents the field. For example, an interpretation session in which UCD experts consider and discuss the alternative UCD-only cluster solution—perhaps guided by follow-up questions from implementation experts—might offer insights into how UCD strategies could best be conceptualized and defined to maximize alignment with implementation science.

## Conclusions

Implementation science and UCD offer complementary approaches with several key points of interdisciplinary alignment. It may be ideal for implementation and UCD experts to work side-by-side to execute strategies from trans-discipline clusters, but work sequentially or in parallel for strategies from discipline-specific clusters. Yet such collaboration could encounter challenges for a variety of reasons. Experts tended to modestly favor their own discipline in their importance and feasibility ratings, suggesting that multi-disciplinary teams could disagree about how to prioritize various strategies when resources are limited. It will also be important to develop supports for multidisciplinary implementation-design teams, drawing on the growing science of team science [46]. In the future, our research team plans to investigate UCD-focused team science resources (e.g., mentored development programs) and tools (e.g., shared online workspaces) to complement the limited, but growing, offerings of implementation training initiatives [47] and our UCD strategy glossary for implementation experts [22]. Our efforts will be informed by continued analysis of additional data collected from participants in this study. For example, participants also provided rank-order and qualitative feedback about challenges and desired supports for cross-discipline collaboration (see the study protocol [9] for details).

In addition to support for collaboration, other advances will be needed to fully realize the potential impact of aligning implementation science and UCD. One important step will be to continue merging implementation-focused frameworks (e.g., [10, 36]) with frameworks that describe how to design for implementation (e.g., [15, 31]) to provide a more complete account of the levels and processes involved in successful implementation. Such guidance, along with recent efforts to advance causal models of the relations between specific implementation strategies and determinants [48, 49], could help decision-makers prioritize UCD strategies among the numerous other implementation strategies available (i.e., 73 in the ERIC project). It will also be necessary to test the impact of specific UCD strategies on implementation and clinical outcomes (e.g., the impacts of some strategies, such as personas, remain unclear), considering again the need to select strategies that address a given problem [49]. Evaluating cost-effectiveness will also be important, given the potential high costs of incorporating UCD into implementation efforts [50]. Finally, it will be important to consider when researcher efforts involving UCD experts can be made “backwards compatible,” meaning they advance scientific understanding and large-scale impact within the UCD field, and/or “forwards compatible,” in which design strategies are described in sufficient detail to inform future implementation research of relevance. The promising level of alignment between implementation and UCD strategies indicated in this study suggests that such efforts will be worth the advances they can bring in terms of advancing health, wellness, and EBP availability.

## Supplementary information

**Additional file 1:** Reporting guidelines checklist for this study.

**Additional file 2:** Alternate cluster map for clusters dominated by user-centered design (UCD) strategies.

## Data Availability

The datasets generated and analyzed during this study are available from the corresponding author on reasonable request.

## Abbreviations

CFIR: Consolidated Framework for Implementation Research
CSGM: Concept Systems Global MAX
EBP: Evidence-based practice
ERIC: Expert Recommendations for Implementing Change
i-PARIHS: Integrated Promoting Action on Research Implementation in Health Services framework
UCD: User-centered design
