# Supplementary material for: Aligning implementation and user-centered design strategies to enhance the impact of health services: results from a concept mapping study
Source: Implement Sci Commun. 2020 Feb 26;1:17. doi: 10.1186/s43058-020-00020-w (PMC7427975; doi:10.1186/s43058-020-00020-w)
Supplement: Supplementary file 1 — Additional file 1: Reporting guidelines checklist for this study. [file 43058_2020_20_MOESM1_ESM.docx]

**Good Reporting of A Mixed Methods Study (GRAMMS; O’Cathain et al., 2008)**

| Item | Page Reported |
| --- | --- |
| (1) Describe the justiﬁcation for using a mixed methods approach to the research question | p. 10, first paragraph |
| (2) Describe the design in terms of the purpose, priority and sequence of methods | p. 10, first paragraph, through p. 11, first paragraph |
| (3) Describe each method in terms of sampling, data collection and analysis | p. 8, second paragraph; p. 9, second paragraph; pp. 10-12 |
| (4) Describe where integration has occurred, how it has occurred and who has participated in it | N/A – concept mapping is a self-contained mixed method |
| (5) Describe any limitation of one method associated with the presence of the other method | N/A – concept mapping is a self-contained mixed method |
| (6) Describe any insights gained from mixing or integrating methods | pp. 15-20 |

**Concept Systems guidelines for reporting concept mapping (Concept Systems, Inc., )**

| Item | Page Reported |
| --- | --- |
| *A. Essential components of describing GCM data collection* | |
| (1) Description of your participants (who did you invite, who participated and at which phases of the project) | p. 8, second paragraph, through p. 9, second paragraph |
| (2) The focus prompt for the project | N/A – research team generated ideas/concepts for participants to sort and rate |
| (3) Description of how you collected the ideas | p. 10, second paragraph |
| (4) Brief explanation of idea synthesis and the final statement set | p. 10, second paragraph |
| (5) Description of how participants sorted (online or in-person) | p. 10, third and fourth paragraphs |
| (6) The rating scales posed to participants | p. 10, fourth paragraph |
| *B. Essential components of describing GCM Results* | |
| (1) The stress value computed for the multi-dimensional scaling representation of the sort data | p. 12, third paragraph  p. 13, second paragraph |
| (2) The final number of clusters chosen | p. 12, third paragraph  p. 13, second paragraph |
| (3) The story the map told you | pp. 15-19 |
| (4) Discussion of how the ratings overlay onto the conceptual framework. | p. 13, first paragraph through p. 14, second paragraph  p. 15, third paragraph through p. 16, first paragraph |

References

O'Cathain A, Murphy E, Nicholl J. The quality of mixed methods studies in health services research. J Health Serv Res Policy. 2008;doi:10.1258/jhsrp.2007.007074

Concept Systems, Inc. Group concept mapping resource guide. 2015. https://www.conceptsystems.com/GCMRG#Dissemination. Access 21 Sept 2019.
