# Supplementary material for: Aligning implementation and user-centered design strategies to enhance the impact of health services: results from a concept mapping study
Source: Implement Sci Commun. 2020 Feb 26;1:17. doi: 10.1186/s43058-020-00020-w (PMC7427975; doi:10.1186/s43058-020-00020-w)
Supplement: Supplementary file 2 — Additional file 2: Alternate cluster map for clusters dominated by user-centered design (UCD) strategies. [file 43058_2020_20_MOESM2_ESM.docx]

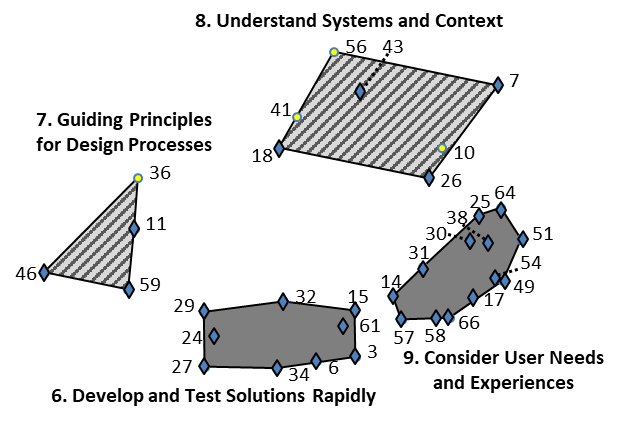


**7. Unnamed new cluster**

*Additional File 2*. Alternate cluster map for clusters dominated by user-centered design (UCD) strategies. The map reflects the product of the UCD experts included in the expert panel (valid response *n* = 21) sorting 66 discrete strategies into groupings by similarity. Circles indicate implementation strategies and diamonds indicate UCD strategies. The number accompanying each strategy allows for cross-referencing to the list of strategies in Table 1. Dark-colored clusters are comprised entirely of UCD strategies, whereas multi-colored clusters are comprised of strategies from both disciplines. Spatial distances reflect how frequently the strategies were sorted together as similar. These spatial relationships are relative to the sorting data obtained in this study, and distances do not reflect an absolute relationship.
